# Photoperiodic and intrinsic circadian regulation of heart rate in *Daphnia pulex*

**DOI:** 10.1098/rsbl.2025.0142

**Published:** 2025-06-18

**Authors:** Timothy J. Hearn, Millicenta Ampiah, Linda King, David Whitmore

**Affiliations:** ^1^ Department of Medical Genetics, University of Cambridge, Cambridge, UK; ^2^ School of Life Sciences, Anglia Ruskin University, Cambridge, UK; ^3^ Australian Institute of Tropical Health and Medicine, James Cook University, Townsville City, Queensland, Australia

**Keywords:** circadian, *Daphnia*, diurnal, heart

## Abstract

*Daphnia* species are well-established models for drug testing. These crustaceans possess a unique myogenic heart similar to mammals and a transparent body akin to zebrafish, making them particularly suitable for cardiac studies. Circadian rhythms have long been identified in *Daphnia* species, with recent reports highlighting photoperiodic responses and circadian gene expression patterns specifically in *Daphnia pulex*. Using biological rhythm analysis and simple automated infrared microscopy, we investigated the heart rate of *Daphnia pulex* to determine whether a diurnal rhythm was present, whether this rhythm was entrained by photoperiod length, and if its control was intrinsic to the heart itself. Our results confirmed the presence of circadian rhythms in the heart rate of *D. pulex*, demonstrating clear photoperiod modulation of entrainment during both diurnal and circadian phases, with these rhythms intrinsic to the heart.

## 1. Introduction

Tissue-driven diurnal regulation of heart rate has been shown in both mice [1] and zebrafish [2]. In the case of zebrafish, we have reported that the circadian clock within isolated larval hearts is capable of driving a circadian rhythm of heartbeat rate, without input from the central nervous system [2]. Circadian rhythms are endogenous, self-sustained oscillations of roughly 24 h that regulate physiology and behaviour to match the diurnal environmental cycles. To demonstrate that a process is truly circadian, one must show that the rhythm persists under constant conditions and can be entrained by a zeitgeber (ZT) such as a light–dark cycle. The zebrafish is an excellent model because it is transparent, and therefore physiological changes can be easily observed *in vivo*. The observation of circadian rhythms in heart rate in fish and mice might indicate either a common feature of myogenic hearts or convergent evolution. To explore this, we have exploited a different model system, the crustacean *Daphnia pulex*. Similar to zebrafish, *Daphnia* is transparent, and the heartbeat can be easily observed under a light microscope. The heart of *Daphnia*, unlike other crustaceans, is myogenic, that is the heart's contractile response originates within the tissue itself [3,4]. The *Daphnia* heart is susceptible to drugs known to affect the human heart, such as caffeine [5], and such pharmacological assays are used worldwide for simple undergraduate laboratory teaching experiments about heart function.

Circadian rhythms have been known for a long time in *Daphnia* species. In *Daphnia magna* long-running rhythms of vertical distribution in the water column were first reported in 1963 [6], and the phototactic response in 1970 [7]. Recently, diel rhythmicity has been reported in *D. pulex* for widespread gene expression in a 12 h photoperiod [8]. Homologues of the core clock genes from *Drosophila melanogaster* have been identified in *D. pulex* [9], and have been demonstrated to have both diel and circadian rhythmicity [10]. Similar to *Drosophila*, the *PERIOD* gene remains a central component of the clock, with diel vertical migration losing rhythmicity under constant light in *PERIOD* knock-out clones, where the circadian clock is disrupted [11].

*Daphnia pulex* has further been shown to have a wide variety of photoperiodic behaviours, including increased production of male offspring and reduced neonate production in short days [12,13], with short day recognition for sex differentiation found to be under clock control [11]. ROS production has a different diurnal profile in short versus long days, and this is associated with the changes in phase of antioxidant gene expression levels [14]. Circadian clock gene expression appears to demonstrate considerable plasticity to photoperiod, with natural populations originating from ephippia under short day length showing uniform gene expression, but populations from summer and autumn showing heterogeneity, with altered expression levels when analysed under long day photoperiods [15], indicating an ongoing adaptive shift driven by changing photoperiods.

In this study, we looked to see whether a key physiological process, heart rate, had a diel rhythm in *D. pulex* and whether entrainment was modulated by photoperiod length. Modulation of circadian entrainment by photoperiod is demonstrated when the phase of the free‐running rhythm synchronizes to imposed light–dark cycles—shifting predictably with changes in daylength—and these phase changes persist as after effects when the rhythm continues to oscillate with a near-24 h period under constant conditions. We hypothesized that we should see this following entrainment to different photoperiods, and that if heart rate is intrinsic to the heart, we would see a free-running circadian rhythm in isolated hearts. Here, we demonstrate for the first time that circadian rhythms are intrinsic to the heart of even simple metazoan, such as *Daphnia*.

## 2. Results

**Figure 1.**
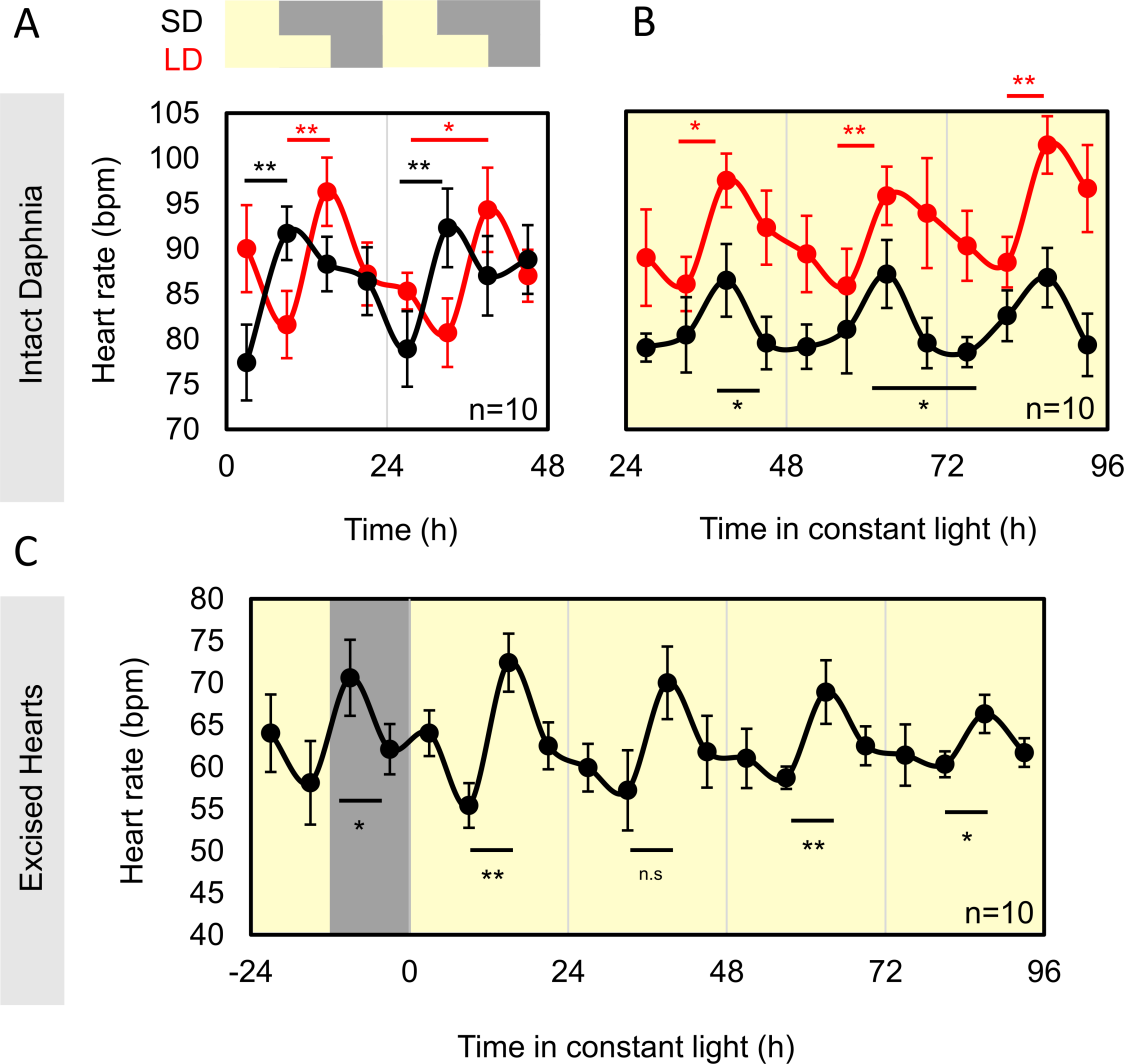
Diurnal and circadian oscillations in heart rate of *D. pulex*. (A) Diurnal heart rate of *D. pulex* female adults under either long day 16 h light : 8 h dark (red) or short day 8 h light : 16 h dark cycles. (B) Free-running circadian rhythms of *D. pulex* female adults after entrainment to either long (red) or short (black) day photoperiods. (C) Entrained diurnal and free-running circadian rhythm of isolated *D. pulex* hearts entrained in 12 h light : 12 h dark cycles. Hearts were extracted from 20-day-old females 48 h before entry into constant light, and counting during the preceding light–dark cycle was performed, followed by 4 days in constant light. On the *x*-axis, 0 indicates the onset of constant light conditions, with −24 to 0 showing the preceding light–dark cycle. Ten 20-day-old individuals were used for each experiment and kept in 96-well plates at 18°C in a light- and temperature-controlled incubator. All *Daphnia* were fully reared in the photoperiod of entrainment indicated. Statistically significant differences between peak and trough heart rate within the same diurnal or subjective cycle are indicated with a coloured bar. Significance levels are denoted as follows: *p < 0.05; **p < 0.01. Significant differences were tested using a paired *t*‐test, except for the comparison between ZT57 and ZT63 in long days (panel B), which was analysed using the Wilcoxon signed-rank test due to non-normal data distribution. All free-running rhythms were found to be rhythmic using JTK cycle (*p* < 0.001).

*Daphnia pulex* were reared in either long (16 h) or short (8 h) photoperiods at 18°C, and heart rate was quantified every 6 h under infrared illumination. The low growth temperature keeps baseline heart rate sufficiently reduced that small differences might be observed. Heart rate was found to be oscillated over the course of 2 days in both long-day and short-day photoperiods, with clearly discernible phase differences (figure 1A). In both long days and short days, heart rate reached peak levels at the beginning of the night, and then declined reaching a minima during the day.

Having established that there is diurnal difference in heart rate with entrainment that can be modulated by photoperiod, we looked to see if the rhythm persisted in constant light, and addressed the hypothesis of whether the changes in phase seen in light–dark cycles would persist as after effects in free-running rhythms. Using the simple automated infrared microscopy system we made for our previous study [2], we were able to measure rhythms from individual *Daphnia* kept in 96-well plates (figure 1B). Heart rate oscillated with a circadian rhythm for three free-running circadian cycles in constant light (JTK: SD, *p* = 0.00001; LD, *p* = 0.00008), with similar period estimates on average data using fast Fourier transform–nonlinear least squares (FFT-NLLS) for *Daphnia* previously entrained to long days (23.24 h) or short days (23.07 h). We saw that peak heart rate continued to be timed to the subjective night, although in both photoperiods this now coincided with the same time points (at 39, 63 and 87 h of constant light). However, the nadir of rhythms was phased differently, with short day entrained *Daphnia* reaching a nadir around subjective dawn, and long day around subjective dusk. FFT-NLLS analysis of the free-running rhythms gave free-running phases of 16 for short day and 19 for long day entrained *Daphnia*. Thus, there is a difference between the phase of circadian rhythm when entrained under different photoperiods.

Finally, we looked at whether control of the diurnal and circadian rhythm in heart rate was endogenous to the heart itself, as in zebrafish, or stimulated by signals from elsewhere in the organism (figure 1C). We identified a method to extract functional hearts from adult *Daphnia*, based on previous work with zebrafish larva. Extracted hearts placed in 96-well plates continued to beat regularly with a circadian pattern (JTK: *p* = 0.00026), with mean free-running FFT-NLLS period estimate of 24.6 h. These experiments were performed with *Daphnia* grown under 12 h photoperiod entrainment conditions. The rate of heartbeat dropped in isolated hearts, but not much, and continued to beat until the end of the experiment. Isolated hearts had a peak at ZT15 under diurnal conditions, and kept this peak phase when in free-running conditions (FFT-NLLS Phase: 15.02 h).

## 3. Discussion

Thus, we have demonstrated that there are circadian rhythms of heart rate in the model crustacean *D. pulex* that show photoperiod modulation of entrainment in both diurnal and circadian phases, and are intrinsic to the heart itself. The role of the cardiac circadian pacemaker in regulating daily heartbeat rate is not only a vertebrate phenomenon and extends back in evolutionary terms to much simpler organisms, perhaps indicating that it is a feature and not convergent evolution, although this can only be speculation. From an ecological perspective, *Daphnia* perform vertical migration during the day, to avoid predators, returning to their position higher in the water column to feed at night [16]. As heart rate in *Daphnia* is also associated with metabolic rate [17,18], then this could explain the increased heart rate at the beginning of the night, as the movement up and down in the water column is an active swimming process. Having this under circadian, rather than direct light control, ensures that the organism moves prior to environmental stimuli.

Given that *Daphnia* possess a myogenic heart [3], this finding opens up the potential to perform circadian experiments on myogenic hearts in a living organism, with biomedical research applications, that is not subject to restrictions applied to experiments with vertebrates. Additionally, given how frequently *Daphnia* is used in undergraduate teaching this is a timely reminder for academic university staff to check the entrainment conditions of invertebrates used in practical classes.

## 4. Methods

### (a) *Daphnia pulex* growth and maintenance

*Daphnia pulex* were acquired from Blades Biological (Surrey, UK). A single female clone was isolated and cultured for three months to generate a large population of genetically identical clones. *Daphnia* were grown in 5 l tanks in dH_2_O and fed with 3 ml 1% yeast solution (Darwin Biological) every 5 days. Tanks were entrained in light–dark cycles with 100 μmol m^−2^ s^−1^ light provided by a full spectrum LED board (Photon LED, 10151145879) positioned above the tank. Tanks were kept in a temperature-controlled incubator at 18°C (Sanyo Growth Cabinet Model MLR-350).

Female clones with empty brood sacks were transferred to individual 75 cm^2^ culture flasks (Greiner Bio-one) in a temperature-controlled incubator (Sciquip SQ-4650) supplied with either long-day or short-day illumination. When neonates were dropped, the adults were removed and the offspring reared for experimentation in 1 l polycarbonate tanks (techniplast). This process ensured that all individuals used had undergone their entire life cycle in the experimental photoperiod. Twenty-day-old adults randomly sampled from multiple tanks were used for experimentation.

### (b) Heart rate monitoring from adult *Daphnia*

For the *in vivo* heart assays, 100 μl of *Daphnia* culture water was transferred to wells of a black clear bottom 96-well plate (Greiner Bio-one). One *Daphnia* was placed in each well and plates placed in entrainment incubators (Ventario T30) at 18°C temperature and 100 μmol m^−2^ s^−1^ equal mix monochromatic red, green and blue (RGB) LED lighting. Light–dark cycles and constant light treatment were controlled by a mechanical timer (TCM24-XD). Plates were placed on the imaging stage of an inverted compound microscope (TELMU XD-1606), itself housed within the incubator. Infrared illumination for imaging was provided by an infrared 940 mm LED (Pimoroni) placed behind a long pass filter—Optolite Infrared Acrylic 1.5 mm (Instrument Plastics Ltd, Maidenhead, UK). Imaging was performed using a Raspberry Pi NOiR camera attached to a 10× monocular eye piece with a 20× objective lens and controlled using a Raspberry Pi 4. To take all measurements under infrared illumination, the LEDs were turned off for 1 min and infrared imaging performed, in an imaging routine analogous to circadian photon counting, to give identical illumination of images in light and dark periods. Videos were acquired for 12 s using the raspivid module in Raspberry Pi OS and saved as MJPEG. A custom Python script was developed to estimate heart rate from MJPEG video recordings by analysing pixel fluctuations over time using SciPy’s signal processing module (scipy.signal, v. 1.14.1) [19]. Briefly, the script reads the video frame‐by‐frame, computes the total pixel intensity (i.e. the summed colour values) for each frame, and records the corresponding timestamps. To reduce artefacts, the final 10 s segment is selected to exclude focusing noise. The brightness signal is then detrended by removing its mean, and FFT is used to obtain initial estimates of the dominant frequency, amplitude and phase of the heartbeat signal. These estimates are refined using a NLLS fit to a sinusoidal model, and the dominant frequency is converted to beats per minute. This approach is superior to simple peak counting because it models the entire waveform—including partial cycles—thereby reducing errors from noise or missed peaks and providing a more robust heart rate estimate. All code is available from the GitHub repository: https://github.com/comparativechrono/microscoPi.

### (c) Isolation and monitoring of intact hearts

To isolate intact hearts we modified the approach we had taken in previous zebrafish work [2], to include mechanical removal of the *Daphnia* carapace without causing excess trauma that causes the heart to stop beating. Within the first hour after dawn, 20 days adult *Daphnia* were washed for 20 s with 10% NaCl and 0.1% Triton X−100, rinsed twice with dH_2_O and transferred to a 30 mm Petri dish containing Leibovitz-15 medium (Gibco) with 15% FBS (Sigma), 0.05 mg/ml of gentamicin (Gibco) and 1× penicillin–streptomycin (Sigma) (L-15/15% FBS). *Daphnia* were returned to entrainment conditions and incubated in media for 20 min, to allow the media to fill the gut. Individual *Daphnia* were then transferred in 100 μl of media to wells of a clear flat bottomed 96-well black plate. Sterile fine forceps were used to puncture the carapace behind the heart, and the carapace slowly opened up. The protocol then followed analogous steps to the established zebrafish larval heart dissection protocol [20]. Opened *Daphnia* were left for 20 min, during which time the organs dissociated. Assessment of the integrity of *Daphnia* and the number of dissected hearts is important here, and gentle pipetting up and down was used to encourage dissociation if needed. Mechanical manipulation of hearts was kept to a minimum, and as much of the remains of the *Daphnia* were slowly removed to leave tissue debris containing hearts in each well. The contents of each plate were then pooled, and the hearts extracted from tissue debris by pipetting the pooled debris onto a 100 μm nylon cell strainer filter (Falcon, Corning Inc., Corning, NY, USA) placed in a 50 ml Falcon tube. The filter was gently washed with an excess of L-15/15% FBS so that the hearts flow through into the tube. The flow-through was pipetted onto a 30 μm MACS® SmartStrainer filter (Miltenyi Biotec, DE) placed in a 50 ml Falcon tube, the flow through was discarded, and the filter rinsed three times with 1 ml L-15/15% FBS medium to retain the hearts in the filter and remove the smaller tissue debris. To collect hearts, the filter was turned upside down on a 30 mm Petri dish, and three washes of 1 ml L-15/15% FBS medium were performed to remove the hearts from the filter. Dissected hearts were then transferred under a stereomicroscope using a pipette into individual wells of a 96-well black plate (Greiner Bio-one) in 100 μl of media. Wells were verified using an inverted compound microscope to contain only one beating heart, free of other tissue. Intact hearts were imaged using the same approach as for adult *Daphnia*.

### (d) Biological rhythm analysis

Analysis of circadian rhythms was performed with the Biodare2 platform [21,22]. Data covering three free-running cycles were imported to identify rhythmicity using JTK cycle [23] and period analysis conducted using FFT-NLLS [24] and MFourFit [25]. These tools allow for estimation of biological rhythm period using fit to a sinusoidal (cosine-based) model, providing robust estimates of period length, phase and amplitude along with associated confidence intervals, and enabling statistical discrimination between rhythmic and arrhythmic profiles. Phase was calculated using the cosine fit to the analysis window and reported in circadian units. Free-running phase and period values are reported in the text. Statistical analysis was conducted in Python (v. 3.11.11) using SciPy (v. 1.14.1). To assess differences between peak and trough heart rate within the same diurnal or subjective circadian cycle, normality was tested using the Shapiro–Wilk test. Depending on the results, either a paired *t*‐test or a Wilcoxon signed-rank test was applied.

## Data Availability

All data used in this paper are available from the BioDare2 repository for circadian data sharing under CC_BY licence. Description of the data and analysis can be found clearly on each experiment page, and includes full metadata covering hypothesis, biological details, measurement details, time series data and original files, period analysis performed and rhythmicity analysis performed. The intact *Daphnia pulex* heart rate experiments can be found at experiment number 23612 [26] (https://biodare2.ed.ac.uk/experiment/23612) and the isolated *D. pulex* heart rhythm experiment found at experiment number 23676 (https://biodare2.ed.ac.uk/experiment/23676) [27].
